# Empowering Young People with Special Educational Needs to Recognize and Report Child Sexual Exploitation and Abuse: A Mixed-Methods Review

**DOI:** 10.1177/15248380231217047

**Published:** 2024-01-02

**Authors:** Laura E. McMinn, Juliane A. Kloess, Zoe Stephenson

**Affiliations:** 1 University of Birmingham, UK; 2 The University of Edinburgh, UK

**Keywords:** child sexual exploitation and abuse, harmful sexual behavior, special educational needs, mixed-methods systematic review

## Abstract

Young people with special educational needs (SEN), such as intellectual disability and/or autism, are particularly vulnerable to child sexual exploitation and abuse (CSEA). This mixed-methods systematic literature review consolidates findings in respect to how young people with SEN are currently being taught about CSEA in the UK, incorporating empirical and practice-based findings to counteract publication bias. Key databases were searched, and relevant organizations were contacted regarding studies published between 2015 and 2022 (inclusive). Thirteen articles met the inclusion criteria. Of these, 10 adopted a qualitative methodology, and three a mixed-methods approach. The thematic synthesis of the qualitative studies identified the following themes: (a) beliefs and stereotypes about CSEA, vulnerability. and risk have led to young people with SEN being misinformed and misunderstood, and (b) anxiety about the topic of sex and abuse creates polarized views regarding CSEA education in adult guardians of young people with SEN. Themes are discussed in the context of societal biases in respect to vulnerability and risk, and these biases are considered to have a negative effect on how young people with SEN are supported. The findings of this review encourage providers of CSEA awareness education to be mindful of not endorsing harmful stereotypes, and to involve parent–carers as much as possible. This review additionally encourages services and organizations to increase focus on practitioner reflexivity and regular training to counteract potential biases in respect to gender, vulnerability, and risk.

## Introduction

The Independent Inquiry into Child Sexual Abuse (IICSA, 2017) recently published its Final Report, highlighting the alarming extent to which institutions across England and Wales have failed to protect young people from CSEA (IICSA, 2022s). The IICSA was a statutory inquiry for England and Wales, independent of government departments, which afforded it the authority to request any materials and information from institutions that would benefit its investigation.^1^ The Final Report (IICSA, 2022) is a culmination of 7 years of investigative work, examining over four million pieces of evidence and over 6,000 testimonies and stories from victims and survivors, which resulted in a series of recommendations to the UK government (HM Government, 2023). Two key recommendations made by the report relate to increasing the knowledge of young people in respect to CSEA: (a) the first directly refers to young people being in a position of comparative weakness, and, as a result, more vulnerable to experiencing CSEA and disadvantaged in respect to reporting abuse and seeking justice; and (b) the second relates to societal attitudes toward CSEA, and the myths and stereotypes which are commonly held. This review does not detract from the key message that the ultimate responsibility for protecting children lies with the adults around them. It addresses one component of safeguarding young people from CSEA, that which is concerned with empowering young people by enabling them to recognize CSEA and report potential abuse to trusted adults and authorities (IICSA, 2022).

## The Scale and Nature of CSEA in the UK

For this review, the term *CSEA* incorporates *child sexual abuse*, that is, a harmful act occurring: (a) within a relationship of power, (b) where the young person holds a position of inequality and whose vulnerability is exploited, and (c) without true consent (Laird et al., 2022). *Child sexual exploitation*, according to a recent systematic review of 66 studies, is defined as:

An abusive act where an individual or group takes advantage of a power imbalance, to use, force, coerce and/or deceive a child or young person into completed or attempted sexual activity, on or offline: (a) by an offer or actual exchange of unmet needs or wants of the child/young person . . . and/or (b) for the economic or social advantage of the perpetrator or facilitator, (c) irrespective of consent or who initiates or solicitates the contact . . . (Laird et al., 2022, p. 13).

Collapsing the concepts of child sexual abuse and child sexual exploitation into one term, namely *CSEA*, highlights the complex and often difficult-to-spot nature of the abuse (Roberts et al., 2020), incorporates the view that CSEA can be perpetrated by a person of any age, includes *peer-on-peer abuse* (i.e., where child sexual abuse involves other children and/or adolescents as perpetrators; Hackett, 2004), acknowledges that abuse can occur through physical contact or online (IICSA, 2022), and recognizes that victims of CSEA can themselves become perpetrators or facilitators (Laird et al., 2022).

The scale and extent of recorded CSEA across the UK is alarming, for example: (a) Barnardo’s Scotland and the Scottish Children’s Reporter Administration notes that CSEA is an issue across the country, with cases identified in 27 of Scotland’s 32 local authorities (Barnardo’s Scotland, 2020); (b) the Police Service of Northern Ireland crime data for the years 2021/2022 show that almost 60% of recorded sexual offence victims are under 18 years old (Northern Ireland Statistics and Research Agency [NISRA], 2022); and (c) England and Wales have seen a 267% rise in child sexual offences recorded by police from 2013 to March 2020, with contact sexual offences increasing by around 202% within the same period (HM Government, 2021). While these statistics may suggest that society is getting better at responding to signs of CSEA and arresting offenders, the prevalence of CSEA across the UK is likely an underestimate, and there is much room for improvement when protecting the most vulnerable in society. This need is acknowledged by the UK government’s response to IICSA’s Final Report (IICSA, 2022), which outlines how the government is addressing the issues raised and includes reference to raising public awareness of CSEA (HM Government, 2023) to combat harmful stereotypes about CSEA and support adults to respond appropriately when abuse is suspected or disclosed. Lacking in the government’s response, however, is how young people themselves are to be empowered to recognize and report potential abuse.

### The Impact of CSEA

Experiencing CSEA has multiple negative effects that often prolong into adulthood (Hailes et al., 2019; IICSA, 2022), including post-traumatic stress disorder and eating disorders (Hurst, 2021; Reingold & Goldner, 2023), depression and suicidal ideation (Easton et al., 2019; Tsur et al., 2022), problems in adult relationships (Labadie et al., 2018; Stige et al., 2020), and difficulties in education and work (Hardner et al., 2018). Experiencing CSEA is associated with an increased risk for revictimization (Walker et al., 2019) and is itself a risk factor for the perpetration of violence and/or sexual aggression (Krahé & Berger, 2017; Papalia et al., 2018). As such, it is imperative that work is directed to prevent and disrupt this cycle of abuse early on.

### Contemporary Issues and Gender

The nature of CSEA is complex and evolving (IICSA, 2022). Issues which have emerged within the past decade, due to sudden advances in technology, are peer-on-peer abuse and online risks (Barnardo’s, 2016; IICSA, 2022; McAlinden, 2018). Today, young people have ready access to extreme pornographic material through smartphones and computers (Attwood, 2017). They may be bombarded with social media content endorsing harmful stereotypes around sexual violence (Stubbs-Richardson et al., 2018), such as *victim blaming* and *just world* (Lerner, 1980) distortions, which communicate the dangerous idea that a victim of sexual abuse has somehow invited or provoked the abuse. Such messages can impact how young people in general understand relationships, consent, and sex, and differentiate between normal–healthy sexual behaviors and those that are problematic, risky, or harmful (Coy & Horvath, 2018; Sun et al., 2016; Tomaszewska & Krahé, 2018). Contemporary online risks for young people include self-generated child sexual abuse material and perpetrators using online platforms to target young people (Internet Watch Foundation, 2021). The UK government recognizes these modern risks to young people and, as such, have included explicit focus on peer-on-peer abuse and online risks in statutory *Relationships and Sex Education* in England and Wales for mainstream and special educational needs (SEN) schools (Department for Education [DfE], 2019; Long, 2020; PSHE Association, 2020).

When considering gender in the context of CSEA, it is likely that one thinks of a male as perpetrator, and a female as victim. This is unsurprising, as much of the literature is largely filtered “through the prism of victim as female and perpetrator as male” (Cashmore & Shackel, 2014, p. 75). As such, practitioners and professionals may hold unconscious gender biases in respect to vulnerability and risk (Barnardo’s, 2016; Cockbain et al., 2017; Hill & Diaz, 2021), which have been noted to increase vulnerability and complicate disclosure (Hill & Diaz, 2021; Nodzenski & Davis, 2023). Both boys and girls can experience CSEA (Josenhans et al., 2020), and both can demonstrate harmful sexual behavior (HSB) toward their peers (Barnardo’s, 2016). Indeed, it has been suggested that the needs of these children and young people, and the safeguarding responses to meet these needs, are similar (Hallett et al., 2019), and both areas of practice have histories where issues of responsibility and blame have caused stigmatization and attention toward the problematic or risky behaviors of children, rather than their needs and circumstances (Brown, 2004; Chaffin, 2008; Hallett, 2017; McAlinden, 2018). As such, both fields of research and practice may have much to offer when informing how young people may be educated about CSEA.

### Prior Reviews

Literature reviews in respect to CSEA awareness-raising for young people in general point toward a wide range of study designs, many being non-empirical (Rizo et al., 2019; Walsh et al., 2018). For example, out of 13 included studies, Rizo et al.’s (2019) initial searches identified a distinct lack of literature on the topic, which necessitated expanding their inclusion criteria to include “think pieces” (i.e., conceptual articles; p. 31) and literature reviews (Rizo et al., 2019). Their review identified just five empirical studies (published between 2006 and 2014) with focus on educating young people (ages not reported) about CSEA; four of these were of a qualitative nature, and participants were mainly professionals and service providers in the United States (Rizo et al., 2019). Rizo et al.’s (2019) findings recommended that programs should include warning signs of sexual exploitation, disclosing exploitation, and how to have healthy relationships. Walsh et al. (2018) focused on randomized control trials of school-based education programs for young people aged 5 to 18 years (99% of participants were from primary schools), with publication dates ranging from 1980 to 2015 (17 studies were published before 2000). Walsh et al. (2018) identified 24 studies in total, mostly conducted in the United States and Canada. Although the authors concluded that school-based programs are generally effective at increasing knowledge and protective skills (Walsh et al., 2018), much of the literature is outdated and may not apply to young people in the UK in current times.

Taken together, these reviews (Rizo et al., 2019; Walsh et al., 2018) point toward a general lack of literature in this area, and a change over time in how this topic is being examined. This change likely reflects how the issue of CSEA has changed and evolved with an increased understanding of this concept and contemporary issues. For example, many historical programs around child sexual abuse focused on the topic of *stranger danger* (Walsh et al., 2018); however, it is now understood that greater risk comes from inside the child’s social network (McNeish & Scott, 2018). Due to the drastic changes in recent years regarding how CSEA is understood, research relating to educational programs that predates the year 2000 may not be entirely relevant to current times.

### Young People with Special Educational Needs

The term *SEN* is a statutory term used across England, Wales and Northern Ireland to refer to any child or young person who has a learning difficulty, challenge, or disability, which requires special educational adjustments to be made for them (Department for Education [DfE], 2015). The DfE recognizes that young people with SEN may experience developmental delay, and as such includes young people up to the age of 25 (DfE, 2015). Young people with SEN may have needs related to a global intellectual/developmental disability (IDD), a specific learning difficulty, such as dyslexia, neurodevelopmental conditions, such as autism or attention deficit/hyperactivity disorder (ADHD), and/or conditions characterized by emotional or behavioral needs, such as conduct disorder (DfE, 2015). Their social and sexual development is further different to that of their peers, which may make them vulnerable to becoming both victims and/or instigators of sexually problematic or harmful behavior as a result of impairments in their ability to mentalize (i.e., to consider the mental states of self and other) and regulate behavior (Allardyce & Yates, 2018; Crehan & Sperry, 2021; Durrleman, 2020; Franklin et al., 2015; Hackett et al., 2013; van Goozen et al., 2022).

In 2015, Barnardo’s published a report highlighting that young people with SEN are at increased risk of experiencing CSEA due to several factors, including over-protection, social isolation, and society refusing to acknowledge them as sexual beings, which engenders a disbelief that they can be sexually exploited (Franklin et al., 2015). In addition, this is further exacerbated by: (a) communication barriers between them and their adult guardians making it difficult to recognize and report signs of abuse; (b) the adults around them misinterpreting potential signs of abuse; (c) increased dependency on others for their care; and (d) a lack of education around CSEA and keeping safe (National Society for the Prevention of Cruelty to Children [NSPCC], 2022).

### Rationale for Current Review

The IICSA inquiry was established in 2015 (IICSA, 2022). That same year, Barnardo’s published their report highlighting the CSEA risks faced by young people with SEN (Franklin et al., 2015). Almost 8 years have passed since, and it is the aim of this review to explore and examine the current state of affairs of CSEA awareness-raising for young people with SEN since these seminal publications. To the authors’ knowledge, there have been no prior reviews of CSEA awareness-raising programs for young people with SEN. The current mixed-methods review therefore intends to consolidate a range of literature dedicated to CSEA awareness-raising in respect to young people with SEN by answering the research question of “What should providers be mindful of when designing and delivering CSEA awareness-raising programs to young people with SEN?,” and providing an overview of the key aspect providers and facilitators should consider.

## Method

### Ethical Considerations and Reflexivity

Systematic literature reviews have a wide-ranging impact, and it is therefore important that authors reflexively consider ethical issues associated with conflicts of interest and the representation of marginalized groups (Suri, 2020). The ontological orientation of this systematic review is critical realism, and its epistemological position is contextualism. In other words, this review acknowledges an objective reality while recognizing that this reality is altered through: (a) the interpretation of participants within each study, (b) the interpretation of the authors of each study, and (c) the interpretation of the review author(s). The first author has experience of working systemically as part of a multidisciplinary community team providing mental health and well-being support for young people with SEN and ascribes to the *social model of disability* (Oliver, 1983, 1990), which views environments and cultures as disabling, rather than locating the problem within the individual (Oliver, 2004). They have critically reflected on the positioning of the authors of included studies, as the information analyzed in this review has already been refracted through a subjective lens, and have maintained a reflective diary throughout the process, as well as utilizing supervision to consider their impact on the analysis. Due to the potential for publication and search biases (Petticrew & Roberts, 2005), the first author sought out unpublished research by contacting organizations, whose work focuses on CSEA, young people with SEN, and/or young adults with learning disabilities, and through conducting a search using Google Scholar.

### Search Strategy

A systematic search strategy was developed by the first author in consultation with professionals working in the field of SEN and mental health and refined in consultation with a subject matter librarian. The search strategy was initially guided by the *SPIDER* configuration (sample, phenomenon of interest, design, evaluation, and research type), designed specifically to identify qualitative and mixed-methods studies (Cooke et al., 2012). Scoping searches were conducted between July 4, 2021 and February 26, 2022 for the purpose of refining the strategy for each database. During scoping searches, the *R* was dropped due to restricting the relevant research pool, and the terms “child sex* offender” and “child sexual abuse” were removed due to diluting the pool of relevant literature. The terms “commercial sexual exploitation of children” and “CSEC” were not used following scoping searches solely retrieving research conducted outside of the UK. Searches were conducted electronically due to the high probability that relevant articles published within the specified timeframe would be available online. An online search was conducted on March 11, 2022 of the following databases: Europe PMC, EBSCO, Ovid (EMBASE, MEDLINE, PsycINFO, Social Policy, and Practice), ProQuest, SCOPUS, and Web of Science (Core Collection). The search strategy was amended to adhere to the individual syntax requirements of the different databases (see Supplemental Table 1). Search results were exported to EndNote, Version 20 (Clarivate, 2013) for the purpose of screening.

### Ancillary Searches

Additional searches were conducted on March 5, 2022 via Google Scholar, using the key terms “special educational needs,” “child sexual exploitation,” “harmful sexual behavior,” and “(prevention OR education).” The results of these were exported to EndNote (Version 20) under a separate group to the main search. Third sector organizations were contacted between November 6, 2021 and January 6, 2022 to enquire about unpublished and in-press research. In addition to reviewing their websites on November 6, 2021, the following organizations were contacted via email: Sexual Violence Research Initiative, The Children’s Society, Barnardo’s, Center of Expertise on Child Sexual Abuse, IICSA, Respond, Mencap, Ann Craft Trust, British Institute for Learning Disabilities, NSPCC, Reach, National Education Union, Sex Education Forum, National Survey of Sexual Attitudes and Lifestyles, Child Sexual Abuse Journal, Marie Collins Foundation, Lucy Faithfull Foundation, and Safer-IDD (Tizard Center).

### Inclusion and Exclusion Criteria

Specific criteria were applied to the results of the search (see Supplemental Table 2). To be prioritized for inclusion in the review, the age range of the young people with SEN had to be between 13 and 25 years (the age range covered by the term SEN). Due to the limited pool of data, the upper age limit was removed in the abstract screening phase in cases where the phenomenon of interest was evident (i.e., if the findings related in some way to sexual abuse prevention and could be extracted to answer the research question). Finally, the review was inclusive of academic, and gray literature where sufficient data could be extracted (i.e., studies published by third sector organizations, and academic dissertations). The inclusion of gray literature in systematic reviews reduces the impact of publication bias and leads to a more balanced view of the available evidence (Paez, 2017).

### Search Results

**Figure 1.**
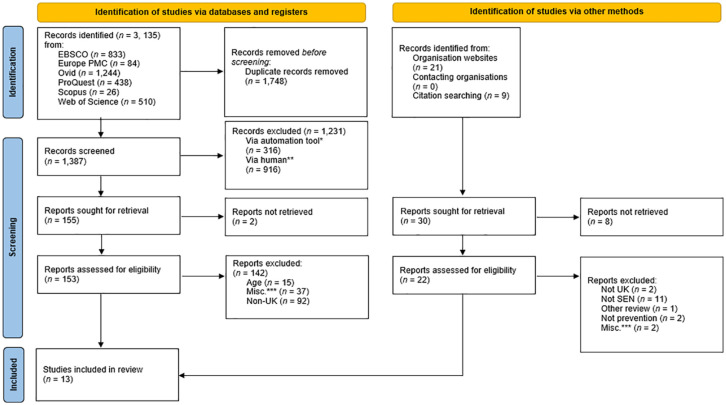
PRISMA diagram. *Note.* Adapted from Page et al. (2021). ^a^Date criteria applied using EndNote. ^b^Title/abstract screening. ^c^Includes conference proceedings, book chapters, psychometric tool evaluations, editorials, other reviews.

The Preferred Reporting Items for Systematic Reviews and Meta-Analyses (PRISMA) diagram for the search results and screening process is provided in Figure 1. Searching the six electronic databases resulted in 3,135 articles. Following the removal of duplicates, 1,387 titles were screened for eligibility in EndNote (this process was undertaken in line with Bramer et al.’s (2016, 2017) guidelines). The abstracts and method sections of the remaining 155 articles were then screened against the criteria. Inter-rater reliability was checked for six abstracts that were chosen at random, which produced consistent outcomes between the first author and their colleague (a Trainee Psychologist). A review of websites of the aforementioned organizations identified 21 articles related to HSB and CSEA awareness and prevention, respectively. No further articles were retrieved via contacting organizations directly. Eight articles returned through a Google Scholar search were not retrievable from the University’s Library Service, and therefore had to be excluded. Abstracts, executive summaries, and methods of additional studies (*n* = 22) were screened according to the inclusion and exclusion criteria. A total of 13 articles (*n* = 10 qualitative; *n* = 3 mixed-methods) were included in the final review, these articles are identified by an asterix (*) in the reference list.

#### Quality Assessment

The full text of each article was assessed against a quality appraisal tool relevant to its methodology (see Supplemental Table 3). All articles were rated by the first author. To counteract this potential bias, 20% of the articles were additionally assessed by a different rater (the aforementioned colleague of the first author), and inconsistencies were resolved through discussion reaching consensus between raters. The first author has maintained a reflexive account of the decision-making process and utilized supervision to discuss quality appraisal. Articles were rated numerically from 0 (not met) to 0.5 (partially met) and 1 (fully met). Due to the vulnerable population and the sensitive topic area, it was considered important to add weight to detailed considerations of ethical issues. Therefore, an additional criterion was added with focus on the acknowledgment and addressing of ethical issues. This criterion was rated numerically from 0 (no ethical considerations provided), through 0.5 (sufficient ethical considerations for published research), to 1 (detailed consideration of ethical issues above that which is typically observed in published research). Any study which achieved a rating of 0 on this criterion was excluded (*n* = 0). The view was taken that applying quality criteria rigidly may exclude valuable findings relating to practice, the methodology of which may not wholly comply with gold-standard reporting regime. Therefore, study quality was not used as an exclusion criterion, and higher quality studies were therefore prioritized in coding and theme development. Quality ratings were given qualitative descriptors of low, moderate, or high, depending on the individual quality score relative to the maximum score.

##### Qualitative Studies

The Critical Appraisal Skills Programme (CASP) qualitative checklist (CASP, 2018), alongside guidelines for scoring provided by Butler et al. (2016), was used to appraise the qualitative articles (*n* = 10). Both raters reached the same ratings for the two independently rated studies. Eight qualitative studies were assessed as being of high quality (Bates et al., 2021; Finlay et al., 2015; Franklin & Smeaton, 2018; Franklin et al., 2019; McElearney et al., 2021; Pryde & Jahoda, 2018; Taylor et al., 2015; Wilkinson et al., 2015), and two of moderate quality (Coleman & Sharrock, 2022; Malovic et al., 2018). The high-quality studies were observed to have clear aims and rationale for qualitative methodology, the findings were presented clearly, and the research was deemed highly valuable. Five of the high-quality studies provided ethical considerations above that which is typically reported in published studies (Franklin & Smeaton, 2018; Franklin et al., 2019; Pryde & Jahoda, 2018; Taylor et al., 2015; Wilkinson et al., 2015). The two studies of moderate quality were distinct in terms of the criteria met. Malovic et al. (2018) did not sufficiently justify the need for a qualitative methodology and was not observed to have analyzed the data rigorously. Coleman and Sharrock (2022) were not observed to have provided detailed considerations of ethical issues, and elements of the design were not adequately justified. It is also worth noting that 4 out of the 10 qualitative studies did not report on the relationship between researcher and participants (Bates et al., 2021; Franklin et al., 2019; McElearney et al., 2021; Wilkinson et al., 2015).

##### Mixed-Methods Studies

The articles utilizing mixed-methods designs (*n* = 3) did not explicitly state which methodology was used; however, the study descriptions indicated toward sequential explanatory. To appraise the quality of these studies, the six central criteria defined by Bryman (2014) were applied, alongside the additional criterion reflecting ethical considerations. Bryman’s (2014) criteria were chosen as they are bespoke to mixed-methods designs (Halcomb, 2019). Of the three mixed-methods studies, two (Franklin & Smeaton, 2017; Roberts et al., 2020) were appraised as high quality and one (Hannah & Stagg, 2016) as moderate quality. None were explicit about the nature of the mixed-methods design; however, they gave sufficient description of the process whereby this could be inferred.

#### Data Analysis

Due to most included studies having used a qualitative design, quantitative data from the mixed-methods studies were *qualitized* (i.e., quantitative findings were converted into qualitative form to be combined with other qualitative data and analyzed qualitatively; Sandelowski et al., 2006; Stern et al., 2020). Thematic synthesis (Thomas & Harden, 2008) was chosen as the most suitable analysis due to its utility in addressing review questions relating to intervention appropriateness (Barnett-Page & Thomas, 2009), and perspectives and experiences (Thomas & Harden, 2008). Familiarization with study findings was achieved through reading and re-reading the results of the included studies. Analysis occurred at both the level of primary data (i.e., quotes from participants) and the interpretations of the study author(s). Primary data were extracted into a summary table alongside information about the study, restatement of findings (Sandelowski et al., 2013), and quality appraisal descriptor (see Supplemental Table 3). Primary data were organized according to the source of data. During this process, two studies were excluded because they did not have extractable relevant data (*n* = 2). Quality ratings were considered during the development of the thematic framework.

The articles were exported from EndNote, Version 20 (Clarivate, 2013) into NVivo, Version 12 (Lumivero, 2017), and the findings of each article were coded line-by-line using an inductive, data-driven method. As recommended by Thomas and Harden (2008), the research questions were temporarily put aside during the initial coding process. Descriptive codes were produced by directly extracting meaning from the quotes or the author’s account of the findings. The codes which contained mainly data from high-quality studies were prioritized when developing themes. The first author looked for similarities and differences across the codes and grouped these into a hierarchical structure, with new codes being created to capture the meaning of grouped codes. This process resulted in five initial themes. Developing themes were discussed with the supervisory team, refined, and checked against *restatements of findings* (Sandelowski et al., 2013) to ensure that the final themes were rooted in the results of each included study. These themes were then interrogated in respect to the developing patterns, and the implications of these patterns (Braun & Clarke, 2021), alongside how they answered the research question. This process resulted in two core themes. A topic summary was compiled to summarize key recommendations for CSEA awareness raising programs (see Supplemental Material).

## Results

### Overview of the Studies

Please see Table 1 for a summary of the critical findings, and Table 2 for implications for practice, policy, and research. The 13 articles included in the review are summarized in Tables 3 and 4 (see Supplemental Material).

**Table 1.** Summary of Critical Findings.

Critical finding	Description
Views of young people are not adequately represented in the literature.	Most studies included views and experiences of parent–carers and the paid support workforce, with fewer studies obtaining the views of young people themselves, and nil studies directly measuring the impact of programs or teaching on the knowledge, attitudes, and/or behavior of young people with SEN.
Young people with SEN are currently disempowered by the lack of provision.	Parent–carers and practitioners pointed toward gaps in policy, a lack of services, and cuts to funding. Few young people understood what CSEA was until they encountered support services and had already experienced harm. Many young people were dissatisfied with education they had received about CSEA, and this was linked with increased vulnerability through to adulthood.
Recommendations for CSEA awareness and prevention teaching/programs.	The results present detailed suggestions in respect to program content, facilitator factors, and how material should be delivered. The use of specific and concrete language, for example, *good touch* and *bad touch*, is recommended.
Practitioner biases around risk and vulnerability in respect to gender.	Practitioners and parent–carers tended to perceive boys as potential instigators of abuse, while viewing girls as victims. Girls were over-represented in the study examining CSEA services.
Myths and stereotypes around CSEA and HSB negatively impact young people with SEN.	Parent–carers indicated anxiety around talking to their children about CSEA and healthy relationships, fearing that this may lead to increased risk. Young people and practitioners expressed concerns about distressed behavior being viewed by society as challenging, leading to missed opportunities for protection and support.

*Note.* CSEA = child sexual exploitation and abuse; HSB = harmful sexual behavior; SEN = special educational needs.

**Table 2.** Summary of Implications for Practice, Policy, and Research.

Field	Implications
Practice	– Teaching around CSEA awareness and prevention should be multi-modal, contain explicit focus on CSEA, include reference to online risks and peer-on-peer issues, and use concrete language. – Programs should be creative in their design and delivered in a meaningful way for young people with SEN. – Practitioners delivering teaching or programs to raise awareness of CSEA should receive adequate training and supervision to increase their confidence in discussing such issues. – Parent–carer involvement is vital.
Policy	– Local authorities could consider pooling resources for services supporting those affected by CSEA, and those demonstrating HSB as the needs, interventions, and safeguarding responses are similar. – Services supporting those affected by CSEA and those demonstrating HSB should prioritize staff training and reflective practice to mitigate the impact of perceptive biases around risk and vulnerability in respect to gender.
Research	– Future research should explore how young people with SEN experience CSEA awareness and prevention programs. – Research should seek to evaluate CSEA awareness and prevention programs in terms of changes to knowledge, attitudes, and/or behavior, and whether such programs have any lasting impact.

*Note.* CSEA = child sexual exploitation and abuse; HSB = harmful sexual behavior; SEN = special educational needs.

#### Data Collection

Six studies (Bates et al., 2021; Finlay et al., 2015; Franklin & Smeaton, 2018; Pryde & Jahoda, 2018; Taylor et al., 2015; Wilkinson et al., 2015) used data from semi-structured interviews, two used a blend of interviews and focus groups (Franklin et al., 2019; McElearney et al., 2021), one used data extracted from textual records of professionals’ meetings (Coleman & Sharrock, 2022), one (Malovic et al., 2018) used information obtained via an expert-consensus methodology, and one used online surveys (Franklin & Smeaton, 2017). Data were analyzed thematically (Bates et al., 2021; Coleman & Sharrock, 2022; Finlay et al., 2015; Franklin et al., 2019; McElearney et al., 2021), via inductive frameworks (Taylor et al., 2015; Wilkinson et al., 2015), and Interpretative Phenomenological Analysis (Pryde & Jahoda, 2018; Wilkinson et al., 2015). One study (Malovic et al., 2018) described the development of a group intervention, whereby findings were largely in respect to facilitator and author reflections.

#### Participants

Five studies (Franklin & Smeaton, 2018; Hannah & Stagg, 2016; Roberts et al., 2020; Taylor et al., 2015; Wilkinson et al., 2015) accessed the voice of young people with SEN or disabled adults in some way. Four studies (Bates et al., 2021; Franklin et al., 2019; Pryde & Jahoda, 2018; Roberts et al., 2020) gained the views of family members and informal supports of young people with SEN or disabled adults, and seven studies (Coleman & Sharrock, 2021; Finlay et al., 2015; Franklin & Smeaton, 2017; Malovic et al., 2018; McElearney et al., 2021; Roberts et al., 2020) gained the views and opinions of the paid support workforce, school staff, and/or professionals.

#### Sample Characteristics

Across the 13 studies, data from approximately 403 participants were included. Three studies (Coleman & Sharrock, 2021; Franklin & Smeaton, 2017; Malovic et al., 2018) were non-specific regarding their total number of participants (e.g., referring to services or local authorities as respondents), and it is therefore suggested that the true number of participants is likely higher than reported. From the information provided in the studies, participants were approximately 73 individuals with SEN (69 young people and 4 young adults), 63 family members, and 263 paid support workers or professionals. Most studies (*n* = 10) focused on school-aged young people (Finlay et al., 2015; Franklin & Smeaton, 2017, 2018; Franklin et al., 2019; Hannah & Stagg, 2016; Malovic et al., 2018; McElearney et al., 2021; Pryde & Jahoda, 2018; Roberts et al., 2020; Wilkinson et al., 2015). Three studies focused on disabled adults, with one asking participants to recall their childhood experiences (Bates et al., 2021; Coleman & Sharrock, 2022; Taylor et al., 2015). A range of needs and conditions were represented, including autism (Franklin & Smeaton, 2017, 2018; Hannah & Stagg, 2016; Pryde & Jahoda, 2018), intellectual disability (Bates et al., 2021; Coleman & Sharrock, 2022; Finlay et al., 2015; Franklin & Smeaton, 2017, 2018; Franklin et al., 2019; Malovic et al., 2018; Pryde & Jahoda, 2018; Taylor et al., 2015; Wilkinson et al., 2015), and disabled and/or D/deaf (Taylor et al., 2015). Two studies referred to participants as having SEN or being recruited from special schools, citing a range of individual needs (McElearney et al., 2021; Roberts et al., 2020).

#### Gender and Ethnicity

The studies with focus on interventions that followed experiences of abuse had a higher proportion of female participants (Franklin & Smeaton, 2018; Taylor et al., 2015), while the study with focus on participants with autism had a higher proportion of males (Hannah & Stagg, 2016), and the studies which sought the views and experiences of parent–carers regarding their disabled children of any gender mainly managed to recruit mothers of sons (Bates et al., 2021; Pryde & Jahoda, 2018). Six studies reported including participants and service users from minority ethnic backgrounds (Coleman & Sharrock, 2022; Franklin et al., 2019; Franklin & Smeaton, 2017, 2018; Taylor et al., 2015; Wilkinson et al., 2015), one study did not formally collect data relating to participants’ ethnicities, instead providing the author’s assumption of participant ethnicity (Bates et al., 2021), and seven studies did not report data on the ethnicity or cultural identities of their participants (Finlay et al., 2015; Hannah & Stagg, 2016; Malovic et al., 2018; McElearney et al., 2021; Pryde & Jahoda, 2018; Roberts et al., 2020).

#### Geographical Location

Samples were reported as drawn from across the UK (Bates et al., 2021; Franklin & Smeaton, 2017, 2018; Franklin et al., 2019; Taylor et al., 2015), the North of England (Coleman & Sharrock, 2022), the South-East of England (Finlay et al., 2015), Cambridge (Hannah & Stagg, 2016), England (Malovic et al., 2018), Northern Ireland (McElearney et al., 2021), Scotland (Pryde & Jahoda, 2018), England and Wales (Roberts et al., 2020), and London (Wilkinson et al., 2015). Participants were recruited from various sources, including social care providers, advocacy groups, schools, colleges, and statutory and non-statutory services. All studies utilized purposive and opportunity sampling, with one (Franklin & Smeaton, 2017) using an additional snowball recruitment method, and one (Malovic et al., 2018) an adapted Delphi method.

### Synthesis of Findings

**Figure 2.**
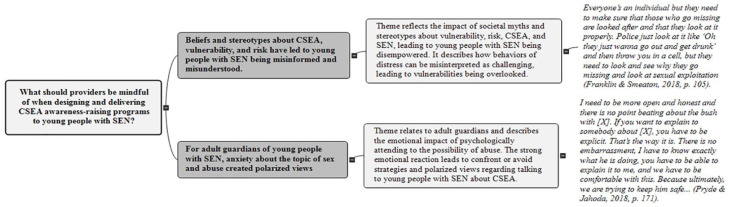
Themes and illustrative quotes.

Synthesized findings from the current literature around CSEA awareness-raising for young people with SEN produced two overarching themes (see Figure 2).

#### Theme 1: Beliefs and Stereotypes About CSEA, Vulnerability, and Risk have led to Young People with SEN Being Misinformed and Misunderstood

This theme reflects the myths and stereotypes that were present in the primary data and connects these with the perceptions reported by young people in the studies. It highlights how young people with SEN reported feeling misinformed about CSEA, how myths and stereotypes about vulnerability and risk may cause young people with SEN to be misunderstood by wider society, and how young people’s behaviors of distress can be misinterpreted by society as challenging, which presents a barrier to effective safeguarding. This theme is important for providers to consider as it highlights key stereotypes which limit opportunities for individuals to disclose abuse, and lead victim/survivors to experience negative self-perceptions.

Of relevance to note here is that just five studies accessed the views and experiences of young people with SEN or disabled young adults (Franklin & Smeaton, 2018; Hannah & Stagg, 2016; Roberts et al., 2020; Taylor et al., 2015; Wilkinson et al., 2015). The lack of representation of young people with SEN in the relevant literature reflects a general lack of the voice of this marginalized group within the wider literature and reinforces the view that young people hold a position of comparative weakness in society (IICSA, 2022). It is therefore unsurprising that the young people in these studies felt misunderstood by others (Franklin & Smeaton, 2018; Hannah & Stagg, 2016; Taylor et al., 2015; Wilkinson et al., 2015), and reported there to be a lack of sufficient education around important safeguarding topics such as CSEA (Franklin & Smeaton, 2018; Hannah & Stagg, 2016; Wilkinson et al., 2015). Many young people with SEN expressed dissatisfaction with the education they had received about healthy relationships and keeping safe (Franklin & Smeaton, 2018; Hannah & Stagg, 2016): “I feel like I’ve been given the tools, but I just didn’t use them because they weren’t clear enough” (Hannah & Stagg, 2016, p. 3682). This quote is from a young person with autism and is reflective of the unique needs of this population. Through the description of education concepts as *tools*, it conjures an image of a toolbelt or -box, from which young people can pick preconstructed responses or script for particular social situations. The young person in the quote points toward not having the right tool to fit the specific circumstance, and this reflects a common difficulty for people with autism in terms of abstracting knowledge to different contexts (Zalla & Korman, 2018).

The larger mixed-methods study by Roberts et al. (2020) reported that many young people were unable to define CSEA or identify how to keep themselves safe, or indicated a lack of knowledge of CSEA as a factor which increases vulnerability (Franklin & Smeaton, 2018): “They should teach kids what it [CSEA] is and what they can do to make sure it doesn’t happen to them” (Franklin & Smeaton, 2018, p. 105). This quote, by a young person in receipt of support from a specialist CSEA service, is terse, clipped, and definitive. It lays blame on wider society for failing to protect children and indicates anger fueled by a sense of injustice, while emphasizing the importance of educating young people about CSEA as one component of safeguarding, with the core responsibility lying with the wider system.

Young victims/survivors of CSEA pointed toward societal perceptions of behavior viewed as challenging, disruptive, or risky, and how these can present a barrier to considering whether CSEA may be at its root (Franklin & Smeaton, 2018; Taylor et al., 2015), leading to inadequate safeguarding: “The social workers should have thought why I was always so angry, why I was always behaving badly to the foster parents” (Taylor et al., 2015, p. 14). In the quote, the adult woman identifies her *bad* behavior as a product of the abuse she was experiencing, echoing the Positive Behavior Support (PBS) theory that all behavior happens for a reason (PBS Academy, 2019), and emphasizing the importance of looking beyond the surface when faced with behaviors which challenge. Misinterpreting and labeling a young person’s behaviors of distress as *challenging* leads to adult guardians and professionals inaccurately placing choice and control within the child:

Everyone’s an individual but they need to make sure that those who go missing are looked after and that they look at it properly. Police just look at it like ‘Oh they just wanna go out and get drunk’ and then throw you in a cell, but they need to look and see why they go missing and look at sexual exploitation (Franklin & Smeaton, 2018, p. 105).

Here, the young person refers to young people who *go missing* as potential victims of sexual exploitation. Of interest to note is that this was a common theme referenced by victim/survivors of CSEA (e.g., Franklin & Smeaton, 2018; Taylor et al., 2015). This is important for providers and those working with young people with SEN to bear in mind when working with this vulnerable population, as going missing may therefore be a risk factor for CSEA or could be an indicator of a young person being exploited or abused. The above quote additionally illustrates how organizations which are established to safeguard individuals can misjudge a young person’s behavior, and how the child’s behavior can be wrongly viewed as consensual (Laird et al., 2022). Such representations are widely reported by victims/survivors of abuse (Kennedy & Prock, 2018; Lemaigre et al., 2017) and may have the unfortunate consequence of individuals not reaching out for help or support (e.g., Taylor et al., 2015):

. . . when I was growing up—over 10 years—I always thought, it was my fault because I didn’t know. At the start, I didn’t know but later, I realized he was actually abusing me. I didn’t know how to tell. It was really difficult. I thought it was my fault or what is his fault? Or both, our fault? Then I started to think and panic that I can’t really tell anyone because people will tell me that it was my fault. But really, it was NOT my fault (Taylor et al., 2015, p. 24).

The above quote, by a young man reflecting on their experience of sexual abuse as a child, demonstrates the internalized conflict which occurs for children who have experienced CSEA, the questioning as to whether they were to blame for the abuse, and how this impacts their confidence in reporting or disclosing the abuse. They describe feelings of anxiety around being misjudged by *people* as being to blame for the abuse. The experiences described by participants in this respect emphasize the importance of CSEA awareness programs being clear and explicit about what CSEA is, combatting the harmful phenomenon of *victim blaming*, and communicating how and where young people can go to for advice and support.

Noteworthy here is consideration of gendered perceptions of risk and vulnerability. In respect to the potential gender bias of included studies, studies which recruited participants with experiences of abuse, or identified as at-risk of CSEA, had a higher proportion of female participants (Franklin & Smeaton, 2018; Taylor et al., 2015). This is similar to that reported in the wider literature (Barnardo’s, 2016; Hallett et al., 2019), and is perhaps reflective of a wider bias in society, whereby girls are more likely to be seen as victims and referred to CSEA services while boys may be criminalized for displaying similar behaviors (Barnardo’s, 2016; Cockbain et al., 2017; Hill & Diaz, 2021). In one study, mothers (of young men with diagnoses of autism and IDD) tended toward an anxiety about society’s perception of their sons’ risk, while they themselves maintained heightened awareness of their vulnerability:

I think a few times he has been in the gents [. . .] and he can stare at things and he is not necessarily staring at the guy next to him having a pee [. . .] but a few men have made comments because I suppose they are feeling vulnerable [. . .] I suppose from my point of view its people’s perceptions of what he is doing that is likely to cause more problems than necessarily what he does do (Pryde & Jahoda, 2018, pp. 169–170).

This quote reiterates the feelings in previous quotes by young people themselves, namely that of society, or *people*, misjudging and misunderstanding the young person with SEN and seeing them as dangerous, or their behavior premeditated. The authors placed this quote in the context of “sexual behaviors” (Pryde & Jaoda, 2018, p. 169) when describing the concerns that the interviewed mothers had about people’s responses to their sons’ more socially unacceptable behaviors. The authors noted that this mother described lesser challenges than the other mothers in the study, many of whom spoke of behaviors on the problematic and even harmful end of the sexualized behavior continuum (Hackett et al., 2019). A mother of a daughter (with SEN) interviewed in another study (Franklin et al., 2019) depicted a slightly different scenario:

Physically, my daughter looks like a little lady but what concerns me, she is a crazy child, she is so complex, because she will go to a random person, hug you, kiss you, sit on your knee. That worries me (Franklin et al., 2019, p. 10).

The authors (Franklin et al., 2019) placed the above quote among others describing behaviors which may lead to increased vulnerability to CSEA, with two out of three being from parent–carers talking about their daughters while the other referred to “children on the [autism] spectrum” (Franklin et al., 2019, p. 10). When looking at the above two quotes together, the gendered perceptions of risk are quite striking. The former quote, taken objectively, describes a seemingly harmless behavior within a context which could create harm, and the risk of harm appears more likely to be toward the young man himself than toward anybody else. The latter quote describes unwanted and non-consensual touch toward unknown others, and the behavior itself could be seen as more problematic or even harmful toward others. However, looking at the two quotes, the reader may be more inclined to perceive the young man’s behavior as harmful, whereas the young woman’s behavior may be seen as vulnerable. Providers of CSEA awareness raising programs are therefore encouraged to avoid reinforcing gendered stereotypes of risk and vulnerability, as such stereotypes may lead to young men who have experienced CSEA feeling that they are to blame for the abuse, and feeling unable to disclose or seek help (e.g., Taylor et al., 2015), resulting in practitioners and adult guardians overlooking genuine risks posed by girls (Allardyce et al., 2021).

This theme emphasizes how providers and practitioners should be cautious of the common myths and stereotypes around CSEA, and risk and vulnerability. Particularly prevalent in the included studies were victim blaming and gendered perceptions of risk and vulnerability. Providers may wish to incorporate material into CSEA awareness raising that combats these harmful stereotypes, and practitioners are encouraged to be reflexive in their practice to observe whether their decision-making is being influenced by these common biases and stereotypes.

#### Theme 2: For Adult Guardians of Young People with SEN, Anxiety About the Topic of Sex and Abuse Created Polarized Views

This theme highlights the emotional impact for adult guardians of young people with SEN in respect to psychologically attending to the possibility of abuse, and the resulting confront or avoid strategies used to manage this. It describes how parent–carers, and even some school staff, indicated polarized views as to whether their children should be taught about CSEA, and how professionals have the fortunate position of oversight from the periphery of this system. The theme is important for providers to consider as it demonstrates the strength and power of the system around the young person with SEN, and indicates how best to involve this system when delivering CSEA awareness raising, or indeed other safeguarding programs.

Five studies (Bates et al., 2021; Franklin et al., 2019; McElearney et al., 2021; Pryde & Jahoda, 2018; Roberts et al., 2020) gained the views of parent–carers, family members, and informal supports of young people with SEN or disabled adults, and seven studies (Coleman & Sharrock, 2021; Finlay et al., 2015; Franklin & Smeaton, 2017; Malovic et al., 2018; McElearney et al., 2021; Roberts et al., 2020) included views and opinions of the paid support workforce, school staff, and/or professionals working with young people with SEN. Where studies captured the voices of adult guardians within the immediate microsystem of the young person with SEN, such as parent–carers, there was a strong sense of anxiety portrayed by the primary data. The key areas adult guardians cited as concerns were in relation to *grooming* and online risks (Bates et al., 2021; Franklin & Smeaton, 2017, 2018; Franklin et al., 2019; McElearney et al., 2021; Roberts et al., 2020). The concept *grooming* has received substantial media attention in recent years (e.g., Cockbain & Tufail, 2020; Gill & Harrison, 2015; Lanning, 2017), which has likely provoked anxiety and fear in many parent–carers and could be likened to the *stranger danger* narrative that dominated safeguarding teaching in the past (e.g., Walsh et al., 2018). Such intense focus on this phenomenon may detract from other areas, for example, the evidence that approximately one-third of CSEA reported worldwide is perpetrated by family member(s) (Stoltenborgh et al., 2011). It may be psychologically easier for parent–carers to view danger as something external to the microsystem that can be guarded against (Franklin et al., 2019; Pryde & Jahoda, 2018; Wilkinson et al., 2015). Teachers, school staff, and other professionals, presented a more informed view of risk (Franklin & Smeaton, 2017; McElearney et al., 2021; Roberts et al., 2020), likely due to being detached from the microsystem and having access to safeguarding training and knowledge.

Parent–carers expressed concerns about introducing sexuality-related safeguarding concepts to their children (Bates et al., 2021; Franklin et al., 2019; Pryde & Jahoda, 2018), and the potential for more graphic imagery or materials used within these programs to be frightening for their children (Franklin et al., 2019). Several parent–carers interviewed expressed the belief that such education may encourage problematic or HSB (Bates et al., 2021; Pryde & Jahoda, 2018), a fear which one father justified emotively:

It would be very difficult, dangerous to introduce that [signs for partner and so on] because you are not quite sure where it would go. If you start introducing something, you are really letting the ‘genie out of the bottle’. . .. It’s best for someone like [X] that innocence is maintained (Bates et al., 2021, p. 499).

The authors interpret this quote as reflecting a fear of the young man’s sexuality, with the use of the word *danger* signifying urgency and risk of harm. This was echoed by parent–carers in other studies (Bates et al., 2021; Franklin et al., 2019; Pryde & Jahoda, 2018; Roberts et al., 2020). *Letting the genie out of the bottle* encapsulates the fear of the uncontrollable and suggests that the introduction of sexuality-related education could be catastrophic. *Innocence is maintained* and *someone like [X]* further reflect the infantilization of disabled people that is prevalent in wider society (e.g., Riddle, 2017; Robey et al., 2006). The quote epitomizes the over-protection of disabled people (e.g., Franklin et al., 2015), and how this may be fueled by fear, echoed by a mother of a boy with SEN: “I think we are all over-protective. All of us. That is the only thing you can do. What else can you physically do?” (Franklin et al., 2019, p. 12). Returning to the subject of gender, the anxiety expressed by parent–carers that sexuality-related safeguarding education could increase problematic or HSB was largely made in reference to their male children (Bates et al., 2021; Pryde & Jahoda, 2018), which again highlights a wider gender bias in relation to risk. The fear expressed by parent–carers reinforces the importance of including them, and keeping them well-informed of safeguarding education, and its importance for keeping their children safe.

In response to this felt fear, parent–carers employed different strategies when faced with the prospect of education around CSEA and healthy relationships for their children. The studies portrayed a general avoidance of the subject of sex and CSEA by parent–carers, both expressed by parent–carers themselves (Bates et al., 2021; Franklin et al., 2019; McElearney et al., 2021; Pryde & Jahoda, 2018; Wilkinson et al., 2015), and observed by school staff and professionals (Coleman & Sharrock, 2022; Franklin & Smeaton, 2018; McElearney et al., 2021; Roberts et al., 2020). The following quote, by a parent–carer of a child with SEN captures the inner conflict of parent–carers: “It saved me from having the conversations with him to be honest and it triggered conversations” (McElearney et al., 2021, p. 28). The quote highlights how the parent–carer recognizes the importance of the topic, and of being able to talk to their child about it; however, they are still uncomfortable with broaching the subject and were grateful to the school for opening the dialog. Several parent–carers described a threat-response style of confronting the topic head-on (Franklin et al., 2019; McElearney et al., 2021; Pryde & Jahoda, 2018; Roberts et al., 2020):

I need to be more open and honest and there is no point beating about the bush with [X]. If you want to explain to somebody about [X], you have to be explicit. That’s the way it is. There is no embarrassment, I have to know exactly what he is doing, you have to be able to explain it to me, and we have to be comfortable with this. Because ultimately, we are trying to keep him safe. . . (Pryde & Jahoda, 2018, p. 171).

This quote, by a parent–carer of an adolescent male with a diagnosis of autism and ID, is forceful and direct, and echoes the activist attitudes employed by some mothers of young adults with autism (McMinn et al., 2019). It represents an alternative way of responding to fear through action and approach, rather than avoidance, and could indicate a higher level of resilience or greater access to power resources (e.g., Johnstone et al., 2018) than those parent–carers who tend toward avoidance. Another mother in the same study (Pryde & Jahoda, 2018) indicated toward a balance or compromise between the two poles when talking about her son’s problematic sexual behavior: “In our culture, we are not allowed to [masturbate] [. . .] I can’t, I can’t get him not to do this, because it’s a natural thing, so [. . .] at least I try to teach him not to do it in front of people, especially his brother” (Pryde & Jahoda, 2018, p. 171), suggesting some resilience against perceived judgments from others. Several parent–carers had recommendations as to how CSEA awareness programs could be delivered and were keen to be notified as to what their children were learning about (Franklin et al., 2019; McElearney et al., 2021):

Maybe it would be good to have an outline of what topics, on a weekly basis, so that whenever he came home, I could see what he learnt that day, so that I could either reinforce it or so that I had a real knowledge of what he was doing (McElearney et al., 2021, p. 25).

This quote demonstrates a general lack of knowledge of CSEA by parent–carers (Roberts et al., 2020), and an expressed need from parent–carers to learn more about the signs of abuse (Franklin et al., 2019), which likely feeds back into the anxiety and fear they feel, and may influence teachers and school staff, some of whom prefer to avoid the topic of sex and CSEA (Finlay et al., 2015; McElearney et al., 2021). Interestingly, this was linked to a lack of training and confidence in talking about safeguarding concepts (Finlay et al., 2015; McElearney et al., 2021; Roberts et al., 2020), and highlights the importance of comprehensive mandatory safeguarding training where core topics are repeated regularly and not just in the context of staff induction training (e.g., Roberts et al., 2020).

Confidence and competence in discussing safeguarding topics is important, as open dialogs and affiliative relationships encourage young people to talk openly about their behavior and experiences (Malovic et al., 2018), and can even lead to disclosures of abuse or of material that raises a safeguarding concern (Roberts et al., 2020; Taylor et al., 2015). Alongside confidence in talking about CSEA, practitioners should be knowledgeable about the varying needs within SEN (Franklin & Smeaton, 2017), and therefore be responsive and adaptable when delivering programs (Finlay et al., 2015). One teacher described how a visual activity in a session about CSEA led to a safeguarding referral being made:

I had a child protection possible [disclosure] and that came through with the picture. They had to draw pictures about where they felt safe and where they didn’t feel safe and. . . It was a child protection issue and it was a referral so that was a good outcome in terms of the lesson (McElearney et al., 2021, p. 28).

This quote highlights how a multi-method approach to safeguarding education for SEN is invaluable, as without the use of the non-verbal and less socially demanding activity of drawing, this concern may not have emerged. As a result of the teacher adapting content appropriately to meet the child’s needs, alongside having sufficient safeguarding training and knowledge to respond appropriately, the child was enabled to make the disclosure, and the teacher was able to take steps to ensure the child was safe. Many studies emphasized the importance of collaborative working between schools, professionals, and parent–carers (Franklin & Smeaton, 2018; Franklin et al., 2019; Malovic et al., 2018; McElearney et al., 2021; Pryde & Jahoda, 2018; Roberts et al., 2020; Taylor et al., 2015). Several parent–carers in the study by Franklin et al. (2019) suggested parent–carer workshops to occur alongside CSEA prevention education delivered in SEN schools.

Overall, this theme indicates that providers of CSEA awareness raising programs should be mindful of the influence of parent–carers, and seek to involve them, either through distributing information about the content and delivery of programs, or by inviting parent–carers to a workshop about the program which can incorporate discussion around how to talk to their children about the complex issues raised. Supporting the adult guardians in the system around the young person with SEN to gain knowledge in CSEA awareness and safeguarding education may help to alleviate some of the anxiety experienced by parent–carers, and ultimately reduces avoidance of this crucial topic.

## Discussion

This systematic literature review synthesized evidence from qualitative and mixed-methods studies to generate important factors to be considered when designing and delivering CSEA awareness-raising programs for young people with SEN. Almost all included studies provided information pertaining to the views of parent–carers, school staff, and professionals or paid support workers, regarding how this safeguarding education should be delivered and what topics should be covered. Fewer studies provided data relating to the impact of prevention programs on young people’s knowledge, attitudes, and behavior, or their views and experiences, which reflects the findings of other reviews more broadly in this area (e.g., Rizo et al., 2019; Schaafsma et al., 2015; Schmidt et al., 2020; Walsh et al., 2018), and signifies young people’s position of comparative weakness in society (IICSA, 2022). Key findings generated from the thematic synthesis (Thomas & Harden, 2008) of the data point toward young people with SEN being disadvantaged by harmful stereotypes and biases held by society, and anxiety felt by adult guardians, leading to polarized views as to whether young people with SEN should be taught CSEA awareness. These two themes appear to be linked by the influence of a societal disempowerment of families affected by disability, and are separated by how this influence enacts on young people with SEN themselves, and impacts the adults in their system.

It has been suggested that practitioners hold biases in respect to their perceptions of victims and perpetrators of sexual abuse (Barnardo’s, 2016; Cashmore & Shackel, 2014; Cockbain et al., 2017; Hallett et al., 2019; Hill & Diaz, 2021), which is reflective of the views held by society more generally (IICSA, 2022). This review observed more concern from adult guardians about young men being inadvertent instigators of abusive behavior (e.g., Bates et al., 2021; Coleman & Sharrock, 2022; Pryde & Jahoda, 2018), and young women being victimized (e.g., Coleman & Sharrock, 2022; Franklin et al., 2019). While some parent–carers of young men expressed fear around them being both victims and instigators due to their vulnerabilities (e.g., Pryde & Jahoda, 2018), the general biases were in accordance with the wider literature (Cashmore & Shackel, 2014). Furthermore, girls and young women were over-represented in the study examining the experiences of young people receiving support from a CSEA intervention service (Franklin & Smeaton, 2018), which could suggest that boys’ vulnerability to CSEA is being minimized and over-looked (Cockbain et al., 2017). This bias also applies to intervention and support, as young men presenting with inappropriate sexualized behavior are often vilified in their communities (Hackett et al., 2013), and dealt with via the Criminal Justice System, whereas young women who present with similar behaviors are more likely to be supported by health services (Hickey et al., 2008; Siegel & Fix, 2020). Interventions for young males and females referred to HSB services are often different, with a tendency for the latter to be approached more sensitively, using interventions focused on well-being, and the former being approached through a focus on their behaviors, with pressures to address risky behavior within certain timescales (Barnardo’s Cymru, 2016; Pelech et al., 2021). Despite the cited evidence not being explicitly based on young people with SEN, the findings are likely to be applicable, given that young people who present to such services often have undiagnosed learning needs (Franklin & Smeaton, 2017), and there is a high proportion of young people with SEN in HSB prevalence studies (Bladon et al., 2005; Hackett et al., 2013). A more reflective understanding of practitioner biases may mean that young males referred to HSB services benefit from relationship-focused and trauma-informed work (e.g., Malovic et al., 2018), while genuine risks posed by girls are not overlooked (Allardyce et al., 2021).

Victims/survivors of CSEA, and those receiving support from CSEA services, noted the impact of society viewing their distressed behavior as challenging, leading to missed opportunities to protect them, and increasing their vulnerability (e.g., Franklin & Smeaton, 2018; Taylor et al., 2019). Labeling a behavior as *challenging* attributes the problem to be within the individual, rather than an interaction between the individual and their environment (Weigel et al., 2006), and often results in restrictions, exclusions, and a more punitive response (National Institute for Health and Care Excellence [NICE], 2017). In the case of this review, such labels presented a barrier to adequate safeguarding and allowed young people with SEN to come to harm (e.g., Franklin & Smeaton, 2018; Taylor et al., 2019). A trauma-informed understanding of behaviors which appear problematic, risky, or harmful, views these as communicating distress or an unmet need (e.g., SAHMSA, 2014), and, according to PBS theory, all behavior occurs for a reason (PBS Academy, 2019). As such, institutions and organizations responsible for keeping young people safe may benefit from training in trauma-informed practice and/or PBS in order to reduce punitive responding, and increase their capacity to safeguard young people with SEN.

The young people represented in this review who voiced experiences of stigmatization by society (e.g., Franklin & Smeaton, 2018; Taylor et al., 2019) may have been impacted by the negative attitudes that non-disabled and neurotypical people tend to have toward disabled and neurodivergent people (e.g., Olkin et al., 2019). This points toward a double disadvantage for young people with SEN, given they are already in a position of relative weakness in society (IICSA, 2022) in addition to being affected by implicit and explicit negative attitudes in relation to SEN. For young people with SEN affected by CSEA, this intersection of stigma is further impacted by *victim blaming* phenomena, such as the *just world* beliefs prevalent in social media (Stubbs-Richardson et al., 2018). Adults in this review spoke of online risks as being a concern, indicated within the context of *grooming* by perpetrators. This review argues that it is therefore important to raise awareness of the different types of technology-facilitated sexual violence (Henry & Powell, 2016), as not to risk overlooking other ways in which harm can be caused to young people with SEN when accessing the online world.

It is of note that adult guardians in this review were not unaffected by societal biases. Those in the microsystem around young people with SEN indicated fear and anxiety around topics of sex and abuse, which resulted in polarized responses of avoidance or confrontation of the topic of CSEA (Bates et al., 2021; Finlay et al., 2015; Franklin et al., 2019; Pryde & Jahoda, 2018; Roberts et al., 2020), with professionals offering a detached oversight of this dichotomy (Coleman & Sharrock, 2021; Franklin & Smeaton, 2017). This reflects the marginalization experienced by families of young people with SEN, where professionals hold positions of power external to the immediate microsystem, and therefore appear unaffected by the societal stigma around disability. The antithesis to marginalization and disempowerment is empowerment, and this can be achieved through increasing the power resource (Johnstone et al., 2018) of *knowledge*.

The UK government’s response (HM Government, 2023) to IICSA’s Final Report (IICSA, 2022) notes plans to fund public awareness campaigns to bring CSEA “out of the shadows” (HM Government, 2023, p. 10). This is reassuring as this review points toward open dialogs about CSEA being helpful in preventing CSEA, and supporting those affected to recognize and report abuse. Such campaigns may serve to familiarize families with the topic of CSEA and increase the confidence of adult guardians toward confronting the topic rather than avoidance. Without an open dialog regarding CSEA, and corrective messages from trusted adults in respect to what is healthy versus unhealthy or harmful behavior, young people with SEN may be more susceptible to the distorted views around relationships, sex, and consent that are portrayed online and by the mainstream media (Coy & Horvath, 2018; Stubbs-Richardson et al., 2018; Sun et al., 2016; Tomaszewska & Krahé, 2018), which may in part explain why many young people in this review reported difficulties in understanding what was appropriate and safe in the context with peers (e.g., Coleman & Sharrock, 2022; Franklin & Smeaton, 2018; Hannah & Stagg, 2016; Wilkinson et al., 2015). Similarly, findings from this review point toward a unique vulnerability for young people with autism (Hannah & Stagg, 2016), suggesting that they may need a distinct focus in CSEA awareness education which respects their unique social communication differences.

## Limitations

Similar to previous reviews in this area (Rizo et al., 2019; Schaafsma et al., 2015; Schmidt et al., 2020; Walsh et al., 2018), this review sought to focus on young people. However, the returned literature necessitated expanding its criteria to include the views of invested parties, and therefore involved participants over 18 years. In addition, literature, which evaluates programs in respect to changes in knowledge, attitudes, and/or behavior of young people with SEN, was found to be scarce, if not absent. As such, it is difficult to discern whether prevention programs are effective in this regard, and indeed what young people with SEN think about these programs. Due to the range of methodologies and adaptive approaches, the risk of bias (when considered within traditional hierarchies of evidence) in each included study was high, which naturally impacts the findings presented here. Research methodologies with individuals with SEN and IDD are often idiosyncratic, due to the heterogeneity of this population, the challenges around access, the adaptation of methods for data collection, and the fact that most researchers in this field are practitioners (Schaafsma et al., 2015; Schmidt et al., 2020). However, this need not detract from the value of this review’s findings. The identified lack of research warrants more progressive and experimental views on methodologies, encourages more researchers to explore this area, and calls on practitioners to feel more confident in publishing their work. Finally, it is important to note that safeguarding is the responsibility of the adults around the young person, and that programs aimed at empowering young people represent just one facet of prevention work (IICSA, 2022).

## Implications for Practice and Research

The findings from this review have implications for future research, policy, and practice: (a) further research should seek to explore how young people with SEN experience CSEA awareness and prevention programs, and whether such programs have a lasting impact on their attitudes, knowledge, and/or behavior; (b) providers should ensure that CSEA awareness and prevention programs are creative in their design and delivered in an engaging and meaningful way to young people with SEN. Teaching should be multi-component, with key concepts repeated over time. Content should include explicit focus on CSEA, and reference peer-on-peer abuse and online risks. School staff should receive adequate training and feel confident in delivering programs, and it is recommended that parent–carers are involved in some way; (c) local authorities could consider pooling resources into services which support young people affected by CSEA and those who display HSB, given the needs, interventions, and safeguarding responses are similar for these groups of young people; and (d) services supporting those affected by and at risk of CSEA and HSB should consider increased focus on training and practitioner reflection to mitigate the impact of perceptive biases on referral and intervention practices.

## Conclusion

This review highlighted a paucity of research into how young people with SEN experience CSEA awareness and prevention programs, and whether such programs have any positive and lasting impact. Several suggestions for good prevention programs were provided from multiple sources; however, where programs existed, they did not seem to have been formally evaluated. This review suggests that adult guardians may need additional support and training to facilitate more open dialogs around healthy relationships and CSEA. It is important that society does not shy away from discussions around healthy relationships and sexual abuse, as this inadvertently increases the risk of young people with SEN. More encouragingly, this review has highlighted a breadth of positive practices in creatively delivering preventative education. While this area of research and practice is slowly developing, practitioners, academics, and parent–carers are progressive and adaptive, and wholly invested in supporting the healthy psychosocial development of young people with SEN.

## Supplemental Material

sj-docx-1-tva-10.1177_15248380231217047 – Supplemental material for Empowering Young People with Special Educational Needs to Recognize and Report Child Sexual Exploitation and Abuse: A Mixed-Methods Review Supplemental material, sj-docx-1-tva-10.1177_15248380231217047 for Empowering Young People with Special Educational Needs to Recognize and Report Child Sexual Exploitation and Abuse: A Mixed-Methods Review by Laura E. McMinn, Juliane A. Kloess and Zoe Stephenson in Trauma, Violence, & Abuse

## Notes

1. https://www.iicsa.org.uk/about-us.html.
