# Supplementary material for: Empowering Young People with Special Educational Needs to Recognize and Report Child Sexual Exploitation and Abuse: A Mixed-Methods Review
Source: Trauma Violence Abuse. 2024 Jan 2;25(3):2503–20. doi: 10.1177/15248380231217047 (PMC11155205; doi:10.1177/15248380231217047)
Supplement: sj-docx-1-tva-10.1177_15248380231217047 – Supplemental material for Empowering Young People with Special Educational Needs to Recognize and Report Child Sexual Exploitation and Abuse: A Mixed-Methods Review [file sj-docx-1-tva-10.1177_15248380231217047.docx]

Supplementary Material

# Table 1

*Table Showing Database, SPIDER Configuration Element, and Syntax Used for Each Database*

| **Database** | **Configuration** | **Syntax** |
| --- | --- | --- |
| EBSCO | S | ("Special educational needs and disabilit*" OR "Special needs" OR "Intellectual developmental disabilit*" OR "Intellectual disabilit*" OR (intellectual development* N2 disabilit*) OR "Learning dis*" OR "Developmental dis*" OR (Neurodevelopmental N2 dis*) OR (neurodevelopmental N2 condition) OR Neurodivers* OR ADHD OR "attention deficit hyperactivity disorder" OR (Autis* N2 disorder) OR (autis* N2 condition) |
|  | Pi | (Relationship* N2 sex* AND education) OR "Keep* safe" OR "sex* education" OR "Child sexual abuse and exploitation" OR (child N2 sexual* AND exploit* AND abus*) OR (adolescent N2 sexual* AND exploit* AND abus*) OR (teenager N2 sexual* AND exploit* AND abus*) OR (Harm* N2 sexual*) OR (Inappropriate N2 sexual*) |
|  | D | AB(Questionnaire* OR interview* OR ("focus group" OR "focus groups") OR "observ* study" OR "narrative study" OR "interpretative phenomenological analysis" OR IPA OR "thematic analysis" OR "template analysis") |
|  | E | AB(view* OR experience* OR opinion* OR attitude* OR perce* OR belie* OR feel* OR know* OR understand* OR (behav* N2 change)) OR "self report*" OR (parent N2 report*) OR (teacher N2 report*) OR (carer N2 report*) |
| Europe PMC | S | ("Special educational needs" OR "Special needs" OR "special school*") OR (intellectual* AND (development* OR disab*)) OR "learning disab*" OR ((Neurodevelopment* disab*) OR neurodiver* OR adhd OR "attention deficit hyperactivity disorder" OR (autis* spectrum)) |
|  | Pi | ((Relationship* OR sex OR *sexual*) AND education OR program*) AND (("Child sexual exploitation and abuse" OR "sexual abuse") OR ((Harm* OR Inappropriate) AND sexual* behav*)) |
|  | D | (Questionnaire* OR interview* OR ("focus group" OR "focus groups") OR observ* OR quasi* OR "narrative study" OR "interpretative phenomenological analysis" OR IPA OR "thematic analysis" OR "template analysis") |
|  | E | (view* OR experience* OR opinion* OR attitude* OR perce* OR belie* OR feel* OR know* OR understand* OR behav*) OR ("self report*" OR "parent report*" OR "teacher report*" OR "carer report*") |
| Ovid | S | ((special educational needs or Intellectual or intellectual development*) adj3 disabilit*).mp. OR (learning disorder or learning disability or developmental disorder or developmental disability or neurodevelopmental disorder or neurodivers* or ADHD or autis* spectrum).af. |
|  | Pi | ((relationship adj3 education) or (sex* adj3 education) or (child sexual abuse adj2 exploitation) or (harm* sexual* adj2 behav*) or (inappropriate sexual* adj2 behav*) or problem* sexual* behav*).af. |
|  | D | (Questionnaire* or interview* or focus group* or observ* study or narrative or (interpretative phenomenological analysis or IPA or thematic analysis or template analysis)).ab. |
|  | E | (view* or experience* or opinion* or attitude* or perce* or belie* or feel* or know* or understand* or behav* or ((self or parent or teacher or carer) adj2 report*)).ab. |
| ProQuest | S | noft(("Special educational needs and disabilit*" OR Special near/2 needs) OR (intellectual near/2 disab* OR development* near/2 disabilit* OR "learning disab*") OR (Neurodevelopmental OR Neurodiver* OR ADHD OR "attention deficit hyperactivity disorder" OR Autis* near/2 spectrum)) |
|  | Pi | noft(((Relationship* NEAR/2 sex* AND education) OR "Keep* safe" OR "sex* education" AND "Child sexual abuse and exploitation" OR (child NEAR/2 sexual* AND exploit* AND abus*) OR (adolescent NEAR/2 sexual* AND exploit* AND abus*) OR (Harm* NEAR/2 sexual*) OR (Inappropriate NEAR/2 sexual*))) |
|  | D | ab(Questionnaire* OR interview* OR ("focus group" OR "focus groups") OR "observ* study" OR "narrative study" OR "interpretative phenomenological analysis" OR IPA OR "thematic analysis" OR "template analysis") |
|  | E | ab(view* OR experience* OR opinion* OR attitude* OR perce* OR belie* OR feel* OR know* OR understand* OR (behav* NEAR/2 change) OR "self report*" OR (parent near/2 report*) OR (teacher near/2 report*) OR (carer near/2 report*)) |
| Scopus | S | TITLE-ABS-KEY ( "Special educational needs" OR "Special needs" OR "special school*" ) OR ( intellectual* W/3 ( development* OR disab* ) ) OR "learning disab*" OR ( "Neurodevelopment* disab*" OR neurodiver* OR adhd OR "attention deficit hyperactivity disorder" OR "autis* spectrum" ) |
|  | Pi | TITLE-ABS-KEY(((Relationship* W/2 sex* AND education) OR "Keep* safe" OR "sex* education" AND child W/5 sexual* AND (exploit* OR abus*) OR adolescent W/5 sexual* AND (exploit* OR abus*) OR (Harm* W/2 sexual*) OR (Inappropriate W/2 sexual*))) |
|  | D | ABS(Questionnaire* OR interview* OR "focus group*" OR "case stud*" OR "observ* stud*" OR "narrative stud*" OR "interpretative phenomenological analysis" OR IPA OR "thematic analysis" OR "template analysis") |
|  | E | ABS(view* OR experience* OR opinion* OR attitude* OR perce* OR belie* OR feel* OR know* OR understand* OR (behav* PRE/2 change) OR (self PRE/2 report*) OR (parent PRE/2 report*) OR (teacher PRE/2 report*) OR (carer PRE/2 report*)) |
| Web of Science | S | TS=(("Special educational needs and disabilit*" OR "Special needs" OR "Intellectual developmental disabilit*" OR "Intellectual disabilit*" OR (intellectual development* near/2 disabilit*) OR "Learning dis*" OR "Developmental dis*" OR (Neurodevelopmental near/2 dis*) OR (neurodevelopmental near/2 condition) OR Neurodivers* OR ADHD OR "attention deficit hyperactivity disorder" OR (Autis* near/2 disorder) OR (autis* near/2 condition))) |
|  | Pi | TS=((Relationship* NEAR/2 sex* AND education) OR "Keep* safe" OR "sex* education" OR "Child sexual abuse and exploitation" OR (child NEAR/2 sexual* AND exploit* AND abus*) OR (adolescent NEAR/2 sexual* AND exploit* AND abus*) OR (teenager NEAR/2 sexual* AND exploit* AND abus*) OR (Harm* NEAR/2 sexual*) OR (Inappropriate NEAR/2 sexual*)) |
|  | D | AB=((Questionnaire* OR interview* OR ("focus group" OR "focus groups") OR OR "observ* study" OR "narrative study" OR "interpretative phenomenological analysis" OR IPA OR "thematic analysis" OR "template analysis")) |
|  | E | AB=(view* OR experience* OR opinion* OR attitude* OR perce* OR belie* OR feel* OR know* OR understand* OR behav* OR "self report*" OR "parent report*" OR "teacher report*" OR "carer report*") |

# Table 2

*Summary of the Inclusion and Exclusion Criteria*

| **Inclusion criteria** | **Exclusion criteria** |
| --- | --- |
| Studies with extractable primary research data, utilising a qualitative, quantitative, or mixed methods design; inclusive of programme or service development project reports. | Book chapters (where these referred to primary data, the related published journal articles were searched for), conference proceedings, poster presentations, psychometric tool or assessment evaluations, other reviews, single case designs. |
| Studies conducted within and using participants from the UK, with the exception of studies that were conducted outside of the UK, but where UK participants’ data can be readily extracted. | Studies conducted outside of the UK and where data from UK participants cannot be readily extracted. |
| Studies published from 2015 to 2022 (inclusive). | Studies published before 2015. |
| Studies focused primarily on young people with SEN who are aged between 12 and 25 years. Additionally, studies with focus on disabled adults were included if all other criteria were met and the phenomenon of interest was evident in the study findings. | Studies focused on young people without SEN and those outside of the age range of between 12 and 25 (with the exception of studies focusing on disabled adults if the phenomenon of interest was evident). |
| Studies conducted in the context of abuse prevention and/or those with focus on relationships education incorporating CSEA awareness. | Studies focused on sexual health with no reference to CSEA awareness or abuse prevention. |
| Studies on community samples, inclusive of supported living arrangements. | Studies where the sample represents people detained in a secure hospital under a section order. |

# Table 3

*Summary Table of Included Studies in Respect to Design, Methodology, and Quality Rating*

| Author | Aim (where possible verbatim) | Study Design & EP^[[1]](#footnote-1)^ | Participants & Sample Size | Data Collection Method | Data Analysis | Quality Rating |
| --- | --- | --- | --- | --- | --- | --- |
| Bates et al. (2021) | The study reported here aims to explore how adults with IDD can be supported to form loving relationships, whether sexual or not, from the perspective of family carers, and has the potential to inform further research and the development of support for family carers (p. 493). | Qualitative design, EP not stated but analysis indicates critical realism.  Utilised advisory group of people with IDD to refine focus group guide. | 19 relatives of people (age 15-56; *M* = 29 years) diagnosed with an IDD. 10 people with IDD were aged 15-26 years, 6 aged 30-35y, 3 aged 37y+. 13 people with IDD were male and 6 were female. | Semi-structured, open-ended questions formed focus group discussion guide.  3 in-person focus groups in different locations, and 3 via telephone conference call. | Latent thematic analysis (Braun & Clarke, 2006); | High |
| Coleman & Sharrock (2022) | The project aimed to explore discussions from the Personal Relationship Advisory Group (PRAG) meetings in a community adult learning disability team to influence the development of new relationship resources (p. 402). | Qualitative design, EP stated as critical realist. | Meetings discussed 6 women and 5 men aged between 17 and 61, (*M* = 30 years).  Panel included psychologists, allied health professionals (AHPs) and the subject’s parent/carer. No disabled people present in the meetings. | 12 months of meeting minutes from the PRAG analysed. Minutes not verbatim. 16 minutes in total, where 11 disabled people were discussed. Minutes were typically 1-2 pages long, and meetings lasted approximately 30-40 minutes. | Thematic analysis (Braun & Clarke, 2006). An inductive approach was followed. | Moderate |
| Finlay et al. (2015) | The aim of this study was to carry out a preliminary investigation of how SHE is delivered in practice to young people with intellectual disabilities. We were particularly interested in the barriers that make delivery difficult, the perceived need for such provision, the institutional and social context, and the challenges faced by teachers and community workers (p. 329). | Qualitative design, EP constructivist. | Study was split into two (conversation analysis and interview), data extracted pertained to interview data.  4 participants: 1 youth worker, 1 deputy principal of a special school, 1 outreach worker for sexual health charity, 1 lead of drama organisation for people with ID. | Semi-structured interviews were used which covered range of issues relating to delivering SHE to young people with ID. | Thematic analysis (Braun & Clarke, 2006), process described as both inductive and deductive. | High |
| Franklin & Smeaton (2017) | 1) detail current provision of services for disabled children; 2) explore the views of practitioners, managers and local and national policymakers looking into both enablers of and barriers to good practice; … 4) identify gaps in policy, provision, evidence and research; 5) generate evidence-based recommendations for future developments in this area of work. These aims were defined by the funder (p. 476). | Mixed Methods - integrated design with findings assimilated across data sources; EP not reported but analysis suggestive of critical realism. | 34% of local authorities responded to online survey.  34 professionals working in field of CSEA and/or IDD across UK; 11 from statutory and 23 from voluntary sector.  27 young people (see Franklin & Smeaton, 2018). | Online survey of local authorities; Online survey of CSEA & IDD services;   Semi-structured interviews with professionals; Semi-structured interviews with young people (see Franklin & Smeaton, 2018). | Survey data analysed descriptively; Transcripts and notes from interviews coded inductively; Data were triangulated across collection methods. | High |
| Franklin & Smeaton (2018) | 1) detail current provision to meet the needs of young people with learning disabilities who experience, or are at risk of, CSE; … 3) understand the needs of young people with learning disabilities who are at risk of, or who have experienced CSE, and gather their views on current practice; … 5) generate evidence-based recommendations for future developments in this area of work. This paper presents the voices of the young people interviewed (p. 99). | Qualitative; EP not stated but suggestive of critical realist. | 27 young people (age 12-23) with IDD, 20 female, 7 male. Majority of sample were white British (*n* = 22), and 5 were from minority ethnic backgrounds (*n* = 3 white/Asian and *n* = 2 Black Caribbean).  15 identified as having experienced CSEA and 12 identified as *at risk*. | Flexible semi-structured interview schedule around key themes, participants were not asked directly about experiences of CSEA.  Contextual information was gathered from support workers. | Transcripts and/or detailed notes from interviews were independently coded by two researchers via inductive coding (Ritchie & Spencer, 1994). | High |
| Franklin et al. (2019) | This research was commissioned by NSPCC to better understand how to best engage with parents and carers of disabled children, including children with physical and learning disabilities, and complex communication needs, when it comes to keeping their children safe from child sexual abuse (p. 4). | Qualitative design, EP not stated but suggestive of critical realism. | 30 parents/carers: 24 mothers, 3 fathers, 3 foster carers (female); 2 British South-Asian, 27 British white, 1 *Other*.  Number of disabled young people in families ranged from 1-4; Ages ranged from 4-21, years; majority were male and attended special schools. | 8 participants requested individual interviews (3 in person, 5 via telephone), remaining 22 participants were split into 3 focus groups. | Not clearly defined in report. The presentation of findings is suggestive of a thematic style of analysis. Topics which were beyond scope of research questions were included indicative of inductive approach. | High |
| Hannah & Stagg (2016) | The quantitative element of our study has two main aims and incorporates six experimental hypotheses. The first aim is to identify the feelings and needs that young people with ASD have reflecting their experiences of sex education compared to typically developing young adults. The second aim of the study is to examine the sexual awareness levels of young people with ASD compared to typically developing young adults (p. 3679). | Mixed Methods - a segregated design with configuration of findings, EP reported as critical realism. | 20 ASD individuals and 20 Non-ASD individuals (*N* = 40), aged 12-25. In ASD group: 12 males, 8 females In Non-ASD group: 7 males, 13 females.  Of the 40 participants, 4 individuals (2 male, 2 female) from each group participated in the interviews (*n* = 8). | Self-report questionnaires: Sexual knowledge, experience, feelings and needs scale (SexKen; McCabe, 1999) and Sexual awareness questionnaire (SAQ; Snell et al., 1991).  Semi-structured interviews with 4 participants. | Quantitative data analysed descriptively and inferentially with Bonferroni correction and Bootstrapping methods.  Qualitative data thematically analysed with critical realist framework. | High |
| Malovic et al. (2018) | Led by members of ySOTSEC-ID, the current study details how an adapted intervention model and materials were developed for adolescents with ID and HSB with the aim to address the gaps in evidence-based service provision (p. 50). | Qualitative design; EP not stated but suggestive of crinital realism. | Feasibility study across 6 public health and charity services in England which support young people with ID who have displayed HSB.  Two of these services completed *Keep Safe* groups within study timeframe, within which 7 young people completed the intervention. | 15 ySOTSEC-ID meetings, 14 face-to-face meetings of Keep Safe Development Group (KSDG), 4 teleconferences, 16 meetings of Keep Safe Advisory Group (KSAG) of members of ID community. | Authors critically reflect on development of Keep Safe intervention. | Low |
| McElearney et al. (2021) | Keeping Safe was implemented in five special schools in Northern Ireland in September 2016. This process evaluation aimed to explore the experience and response of staff and parents across these special schools (p. 5). | Qualitative approach and longitudinal design across 2 academic years.  EP not stated but suggestive of critical realism. | 65 individuals (35 teachers, 2 school governors, 7 classroom assistants, 9 parents/carers) from 5 special schools in NI implementing the *Keeping Safe* programme.  School planning and policy documents were also reviewed. | 11 individual and 5 paired semi-structured interviews, and 8 focus groups.  Schools submitted a selection of policy documents relating to the programme. | Thematic analysis based on five-stage framework (Ritchie & Spencer, 1994).  Thematic analysis used to review policy documents. | High |
| Pryde & Jahoda (2018) | This study aimed to address the gap in the literature by undertaking an exploration into the lived experience and views of mothers of sons with ASD and an intellectual disability, in relation to their sons’ sexuality and sexual development (p. 167). | Qualitative; EP not stated but suggestive of interpretive / constructionist. | 5 mothers of sons (aged 16-24 years) with ASD and ID. | Individual semi-structured interviews conducted in-person, 1 interview via telephone at participant's request. | IPA was used following Smith et al.'s (2009) guidelines.  Emergent themes were frequently labelled as direct quotes.  Patterns and connections across themes were explored. | High |
| Roberts et al. (2020) | The Inquiry commissioned this mixed methods research to support its residential schools investigation to understand how residential schools’ safeguarding policies and processes against child sexual abuse operate in practice (p. 15). | Mixed Methods - integrated design; EP not stated but suggestive of critical realism | 15 residential schools from England and Wales  Proforma: 8 special schools participated in the proforma (7 mixed-sex and 1 male school) from across the UK.  Qualitative sample: 16 young people with SEN (9 female, 7 male); 56 staff from SEN school; 9 parents/carers of young people with SEN; 7 professionals from local authorities. | Proforma collected data on safeguarding policies and anonymised info from safeguarding records.  Interviews and focus groups with staff and young people carried out in-person in schools. Telephone interviews with parents.  Interviews guides were flexible and responsive to the language the young person used. | Qualitative data prioritised in report.  Qual. data analysed via Framework in Nvivo.  Proforma data analysed descriptively to explore differences between variables.  Proforma data was used in findings to explore how these linked with qual. data. | High |
| Taylor et al. (2015) | The research aimed to better understand the experiences of abused deaf and disabled children and identify enablers and barriers, in terms of disclosure, recognition and response, within the child protection system (p. 6). | Qualitative design, EP not stated suggestive of critical realism. | 10 deaf and/or disabled people participated.  Ages included 12y (*n* = 1), 13y (*n* = 2), 30-39y (*n* = 1) and 40y+ (*n* = 3).  3 men/boys and 7 women/girls interviewed.  1 participant was from minority ethnic community.  7 participants had experienced sexual abuse.  Participants reported a range of impairments. | Guided conversation (Kvale, 1996) was used as an approach to interview. An interviewer who is native British Sign Language (BSL) user interviewed Deaf participants. Registered BSL/English interpreters interpreted interviews undertaken by the Deaf researcher for those participants who did not use BSL. | Data analysed via inductive coding (Ritchie & Spencer, 1994) and interrogated narratively and cross-sectionally (Mason, 2002). | High |
| Wilkinson et al. (2015) | This research aimed to investigate the development of sexual identity during transition into adulthood for young people with ID from the perspectives of young people with ID and their carers, to provide an enriched, triangulated understanding of this process, and shed light on the nature of the care relationship and its role in sexual identity development during transition to adulthood (p. 95). | Qualitative; EP not stated but suggestive of interpretive / constructionist.  Interview schedule developed in collaboration with service users with ID. | 4 young people (2 female, 2 male) with ID aged 19-22 years.  4 carers (2 male, 2 female), 2 were parents of young person and 2 were paid support workers. | Semi-structured interview, interview focus guide was different for young people and carers. | IPA was inductive and iterative, followed Smith et al.'s (2009) guidelines.  Master themes were organised by identifying patterns between young people’s and carers' accounts. | High |

*Note*. EP = Epistemological position; ID = Intellectual Disability; NI = Northern Ireland.

# Table 4

*Restatement of Findings (Sandelowski et al., 2013) and Exemplary Quotes*

| Author | Restatement of Findings (Sandelowski et al., 2012) | Exemplary Quote (where relevant) |
| --- | --- | --- |
| Bates et al. (2021) | Relatives of people with IDD report several barriers to their relatives developing romantic relationships, including barriers they themselves implement. These barriers reflect the societal infantilisation of people with IDD, and a fear of their sexuality. | It would be very difficult, dangerous to introduce that … because you are not quite sure where it would go. If you start introducing something, you are really letting the ‘genie out of the bottle’…. It’s best for someone like [X] that innocence is maintained (p. 499). |
| Coleman & Sharrock (2022) | PRAG meetings around disabled adults highlighted themes relating to a lack of education and understanding of sex and relationships, and the use of the internet to seek out relationships emerged as a risky area. | X doesn’t appear to recognise the dangers of engaging in a sexual relationship with strangers (p. 407). |
| Finlay et al. (2015) | Facilitators and teachers of sexual health education emphasised importance of creativity and repetition when delivering content to young people with ID, and that material should be delivered in a phased process which expands upon previous concepts learned. | We’ve got the visual thing, the scene (. . .) so people with low understanding would have seen that what I was doing, certainly with the physical stuff was inappropriate (. . .) whereas maybe some of the language, that that might have not got across (p. 331). |
| Franklin & Smeaton (2017) | Professionals working in IDD and CSEA services suggest school-based relationships education should involve CSEA awareness and that professionals must be knowledgeable about both IDD and CSEA to meet the needs of this population. | We need good sex education and awareness work with young people with learning disabilities, especially around boundaries, what is and isn't socially acceptable, how best to work with and empower these young people, and self-protection skills and undertaking safe risks (p. 478). |
| Franklin & Smeaton (2018) | Young people with IDD accessing CSEA services reported a lack of school-based education about what CSEA is, many young people did not realise they had experienced CSEA until they were working with the service. Working with CSEA services helped young people understand what CSEA looks like and increased their self-protective skills. | They should teach kids what it [CSEA] is and what they can do to make sure it doesn’t happen to them (p. 105). |
| Franklin et al. (2019) | Parents of young people with SEN expressed anxiety about their children's vulnerability to exploitation and abuse and were concerned about sexuality education being taught at an appropriate level and delivered in an accessible way. | You can’t shield them so you have to be open and honest with them because you’re making them feel ashamed about their bodies and it’s not going to help the situation (p. 15). |
| Hannah & Stagg (2016) | Young people with ASD are at a disadvantage to their neurotypical peers in respect to knowledge and confidence in sex and sexuality, which may create a vulnerability in terms of becoming a victim or instigator of problematic or harmful sexual behaviour. | [X]: I just keep getting it wrong and just keep messing stuff up. Yea so, it doesn’t really happen in my world.  …  Interviewer: What have these bad experiences entailed?  [X] They’ve entailed me, erm, stalking, I guess. I should just now admit stalking. Just getting on the wrong side, getting jealous. Just yea, feeling ignored (p. 3686). |
| Malovic et al. (2018) | Development and implementation of the Keep Safe intervention was challenging due to lack of resource in services for young people with ID who display HSB. The intervention is strengths-focused and emphasises importance of including parents/carers throughout the programme. Key component for effective delivery is skilled facilitators who create a positive therapeutic climate. | ...Keep Safe manual has a section on facilitator’s group skills, and the sessional activities are designed to provide a model with practice of skills, techniques, and strategies that facilitates the young person’s, and their parent/carers’ engagement and success in treatment (p. 54). |
| McElearney et al. (2021) | School staff found the content of the programme and its interactive resources helpful, however many aspects of content needed adapting to suit varied needs of the young people, and schools experienced difficulty engaging parents-carers as key partners. | But I think that’s what the lessons did. They created a climate in the class where children knew I can actually tell anything in this room. I can say anything I need to in this room and my teacher will listen to me (p. 27). |
| Pryde & Jahoda (2018) | Mothers of sons with ASD and ID felt ill-equipped to manage their sons' challenging sexual behaviours; they were concerned about these behaviours escalating and their sons being vulnerable to becoming victims or instigators of problematic or harmful sexual behaviour. | I need to be more open and honest and there is no point beating about the bush with [X]. … I have to know exactly what he is doing, you have to be able to explain it to me, and we have to be comfortable with this. Because ultimately, we are trying to keep him safe (p. 171). |
| Roberts et al. (2020) | Professionals working with young people with SEN, and the young people themselves, highlighted that prevention work needs to be multifaceted and should not rely solely on young people to seek help. Several topics were noted as beneficial to school based RSE, including staying safe online, having respectful relationships, and how to spot signs of abuse. | You know when you’re best friends and bros? Normally you put your arm all around them; sometimes it could be a good touch, but sometimes they might find it uncomfortable, which is a bad touch (…) I learned about it, like, when I was in Year 3 (p. 41). |
| Taylor et al. (2015) | Disabled children and adults who made disclosures of abuse did so several years after the abuse began because of factors including a lack of understanding that they were experiencing abuse, difficulty communicating experiences, and the adults around them not recognising the signs of abuse. | The social workers should have thought why I was always so angry, why I was always behaving badly to the foster parents (p. 14). |
| Wilkinson et al. (2015) | Young people with ID and their carers highlighted the over-protective power of the ID identity which infantilises the person with ID and restricts opportunities to develop a healthy, adult sexual identity. | I don’t care what anyone else says. I’m just a normal person that wants a girlfriend, like any other teenager in the world, any other grown man would want one (p. 99). |

**Reference**

Smith, J. A., Flowers, P., & Larkin, M. (2009). *Interpretative phenomenological analysis: Theory, method and research*. SAGE.

1. EP = Epistemological position. [↑](#footnote-ref-1)
